# Everybody's Page

**Published:** 1888-04-21

**Authors:** 


### Everybody's Page.

Paralysis may be brought about by cold, a not uncommon form being that affecting one side of the face after sitting with it exposed to a cold draught, which arrests the function of the nerve supplying the muscles of the face, and thus for a time paralyses them.

In 1792, William Tuke, a member of the Society of Friends, was mainly active in instituting the York Retreat for the care and cure of the insane members of the Quaker sect. This was the first asylum instituted in Great Britain conducted on real non-restraint principles.

Sunlight is second only to fresh air in keeping a room pure. The sun's rays have a distinct oxidising power, and destroy many of those low organic forms which are prejudicial to health. In this relation it is interesting to know that the poison of the cobra, though it retains its virulent properties if kept in the dark, loses them on exposure to the sun.

Not much is definitely known as to the localisation of mental functions in different parts of the brain. It is highly probable that such is the case; but science has not as yet been able to produce any substantial proof. The so-called science of phrenology is based on entirely fallacious data. The idea that the skull can be mapped out into small areas indicating the function of the subjacent brain is entirely false. If we look at a brain covered by its tough outer membrane, we see that it is as devoid of irregularities on its surface as a bladder of lard; and if, again, we inspect the inner surface of the skull-cap, we find that if it has any depressions they do not correspond with elevations on its outer table. The phrenological images which are still sold in the shops may be regarded as the fetishes of an extinct faith.

Any sudden strain seems to be specially injurious to the heart. For example, a man for eleven months in the year follows a comparatively sedentary occupation in his office or in his shop, doing very little physical work. He then takes a holiday, and begins it by walking twenty or thirty miles a day on a hilly road or on the hills themselves. His heart, unaccustomed to have such a strain as this thrown upon it, gives way, and he is an invalid for the rest of his days. So much is this the case, that a well-known authority on heart disease says that it would be far better for many men, when they get a holiday, that they should spend it in bed.

Is it possible to make our readers and correspondents understand that, though we make mention of any new drug which appears to us to be really worthy of public notice, The Hospital is not a medium for advertising any and every nostrum which takes the fancy of the credulous. Lately we were asked to call attention to a certain ointment. What its components are we were not informed, but they certainly must be something far finer than the humble British Pharmacopœia can boast of; and it is just possible that we may be throwing away the opportunity of learning one of the lost secrets of the alchemists of bygone days. The work of common ointments is far beneath the dignity of this mystic compound. Its function is, we are told, to relieve "diseases of bones by hastening the removal of dead sequestra, and thus obviating the necessity, in many cases, of amputation and serious operations." Chloroform and carbolic acid, hide your diminished heads! The first can only render amputations painless; the second can only diminish the risk of suppuration and its attendant ills; they will both be superseded, thrown out of work, by this as yet anonymous ointment—if it can only perform what its sponsors promise on its behalf. There is much virtue, much safety, in that *if*.

It has been calculated that the smoke cloud which hangs over London is composed of 60 tons of carbon daily.

It is popularly considered that the first milk that flows from the udder is the best for invalids. This is a mistake, as it has been proved that the last milk is nearly twice as rich in cream, owing probably to the cream rising in the udder from its greater lightness, and being consequently the last to flow. Therefore, if rich milk is wanted, the "strippings" or last milked should be selected.

We may almost say that through statistics we can prophesy the future, for such is the regularity of Nature, and so unvarying are her operations under similar conditions, that though nothing is more uncertain than the time of death of any single individual, yet nothing is less liable to vary than the age at which each thousand of 100,000 children born in any year will have passed away, and so much do varying circumstances tend to balance each other, that the number of accidents on the London streets may be very closely predicted for any day.

Several observers have undertaken the tedious task of counting the number of hairs growing from the scalp. According to Sir Erasmus Wilson the average number is 120,000. But this is greatly influenced by the colour of the hair. Thus another observer has shown that a square inch of scalp will hold 728 flaxen hairs, 678 chestnut hairs, and only 588 black hairs. It follows from this that our blonde beauties have a distinct advantage over our brunettes in so far as the number and fineness of their hairs is concerned. In fact, a lady with a good endowment of flaxen locks combs out and disentangles each time she performs her toilet somewhere between seventy and ninety miles of hair.

The mode of testing ale in vogue 300 years ago was hardly so refined a process as that which we employ at present. It had, however, the undoubted merit of simplicity, and consisted first in tasting the ale. That process having been found to yield satisfactory results, the liquor was then tested to ascertain whether it had been adulterated by the addition of sugar. This test was carried out by the inspector first spilling a quantity of the liquor on a bench, and then, with his leather breeches on, sitting down on it. If sugar had been added, the inspector after a time became so attached to the seat that to quit it was a matter of difficulty. If, however, the ale was pure and free from sugar, he rose from his seat after the lapse of the prescribed time with the usual facility.

A new form of milk adulteration, which appears to be beyond detection, has greatly increased of late. A circular is sent to a dairyman, marked "strictly private and confidential," in which a firm of condensed milk manufacturers offer unsweetened condensed milk in twelve-pound tins at fivepence farthing a pound, stating that one pound, mixed with water, will produce three quarts of "rich, pure, liquid milk," or five quarts for puddings or for infants' use, costing, according to the degree of dilution, three farthings to five farthings a quart, which is much less than the wholesale price of fresh milk. Mr. Lloyd (the chemist of the British Dairy Farmers' Association) appears to have no hope of any means of detecting this form of adulteration being discovered. The strongest objection to it, as a matter of health, lies in the danger of impure water being used for dilution. Besides this, it may be suggested that the public is quite competent to dilute its condensed milk without the intervention of a dairyman, and prefers to buy it under its own name, and not under that of new milk.

